# Isolated splenic tuberculosis detected only on FDG-PET

**DOI:** 10.1259/bjrcr.20150238

**Published:** 2017-04-01

**Authors:** Anuradha Rao, Govindarajan Mallarajapatna, Sharanabasappa Godehal, Swarna Shivakumar

**Affiliations:** ^1^Department of Radiology, Apollo Hospitals, Bangalore, India; ^2^Department of Pathology, Apollo hospitals, Bangalore, India

## Abstract

Tuberculosis (TB) is a well-known problem in developing countries but has shown resurgence in non-endemic populations in recent years. This may be due to increased migration, and is more commonly seen in populations with lowered immunity due to various causes. Isolated splenic TB is extremely rare, especially in immunocompetent patients. In this case report we have described an immunocompetent female, presenting to the physician with fever, without any chest symptoms or weight loss. All microbiological investigations for pyrexia of unknown origin were done, which did not reveal the cause. Imaging modalities including chest radiographs and ultrasound did not reveal any significant abnormalities. Finally, fluorodeoxyglucose (FDG)-positron emission tomography (PET) showed FDG-avid multiple focal nodular lesions (not seen on contrast and non-contrast CT). MRI including diffusion-weighted imaging did not reveal the splenic nodules. PET-directed CT-guided biopsy of the splenic lesions was performed, with histopathology findings suggestive of TB. Atypical clinical and imaging presentations are not uncommon in TB. History of exposure to TB may not be present. Nevertheless, TB should be kept in mind as a differential diagnosis in patients with fever, and extensive search of the source is important. Splenic TB reported in literature to date has been detected by morphological imaging modalities such as ultrasound or contrast CT. Ours is possibly the first case reported in the English literature where FDG-PET has detected the lesions that other imaging modalities failed to show, thus illustrating the role of molecular imaging in the evaluation of pyrexia of unknown origin to localize the site of affection.

## Background

Tuberculosis (TB) is a major health issue worldwide, especially in developing countries. Extrapulmonary TB accounts for 15% of all the TB cases.^1^ Hepatosplenic tubercular involvement is common in disseminated TB and is of either micronodular or macronodular variety.^2^ Isolated splenic TB is extremely rare, the exact incidence or prevalence being difficult to ascertain owing to isolated case reports from different parts of the world.^3^ Delay in diagnosis is expected owing to non-specific clinical symptoms and challenges in imaging.

## Case report

**Figure 1.**
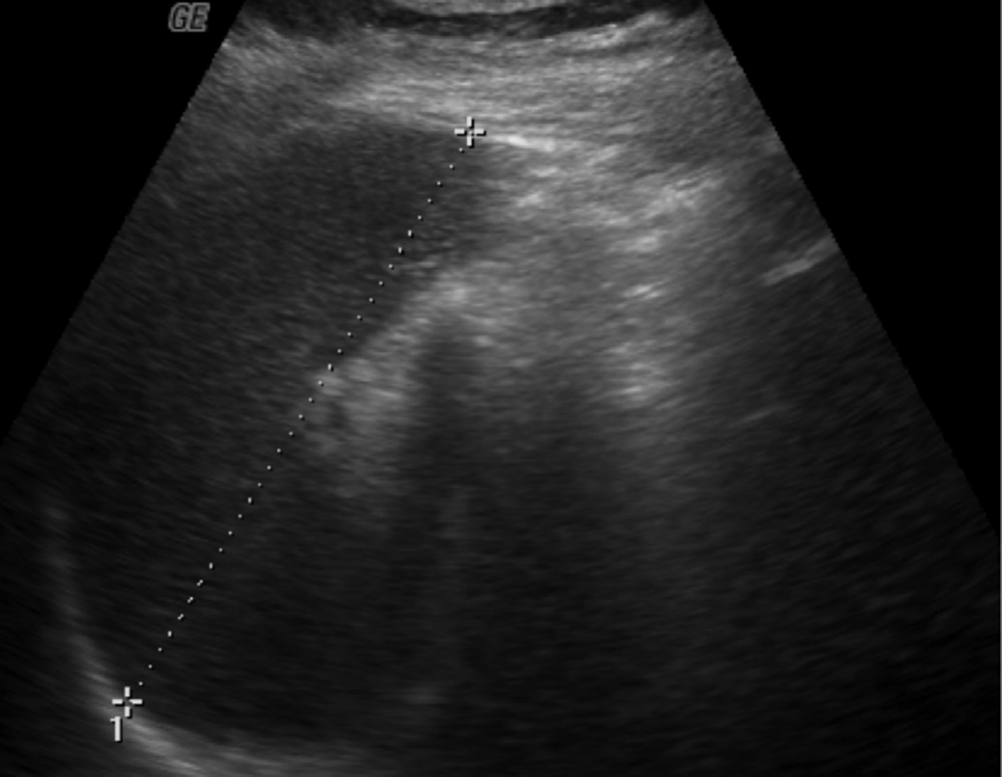
Ultrasound image of spleen showing normal echotexture of spleen without any focal nodular lesions.

**Figure 2.**
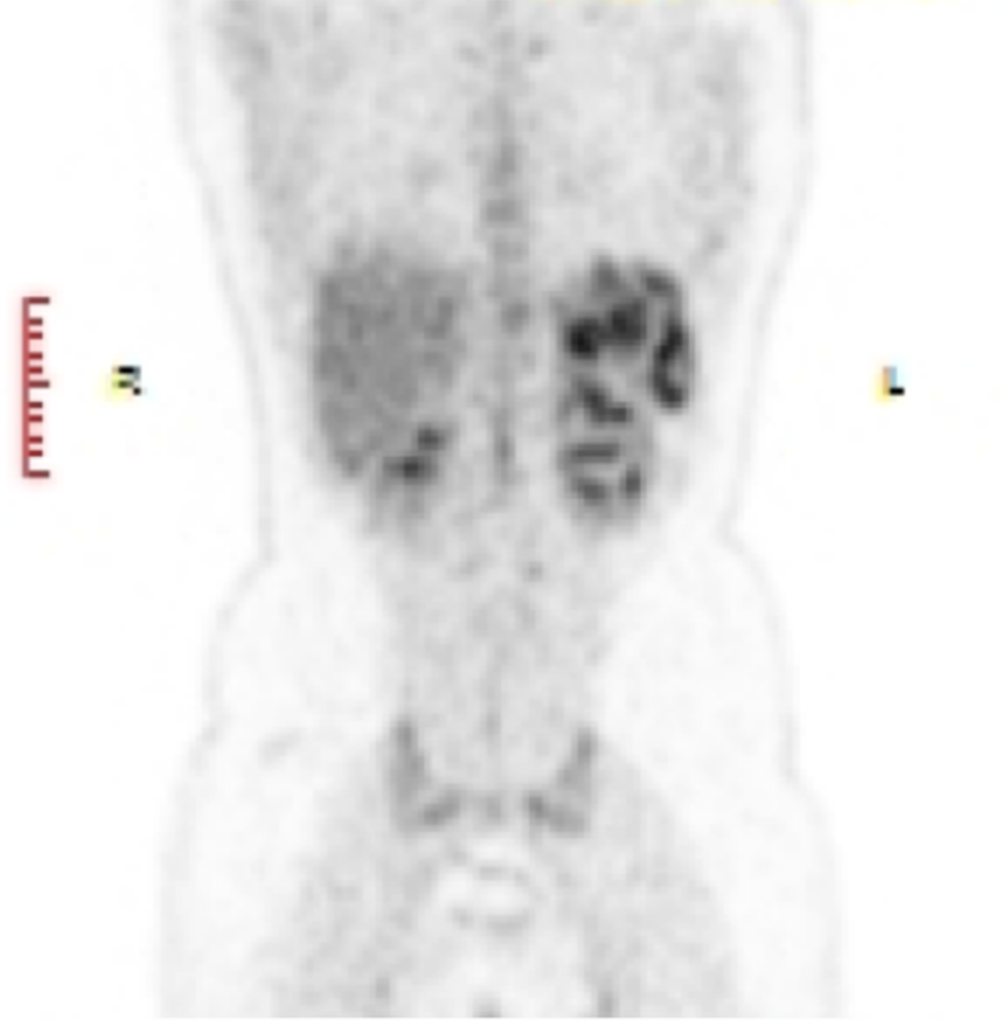
18-Fluorodeoxyglucose-positron emission tomography image showing multiple focal fluorodeoxyglucose-avid nodular lesions in spleen.

**Figure 3.**
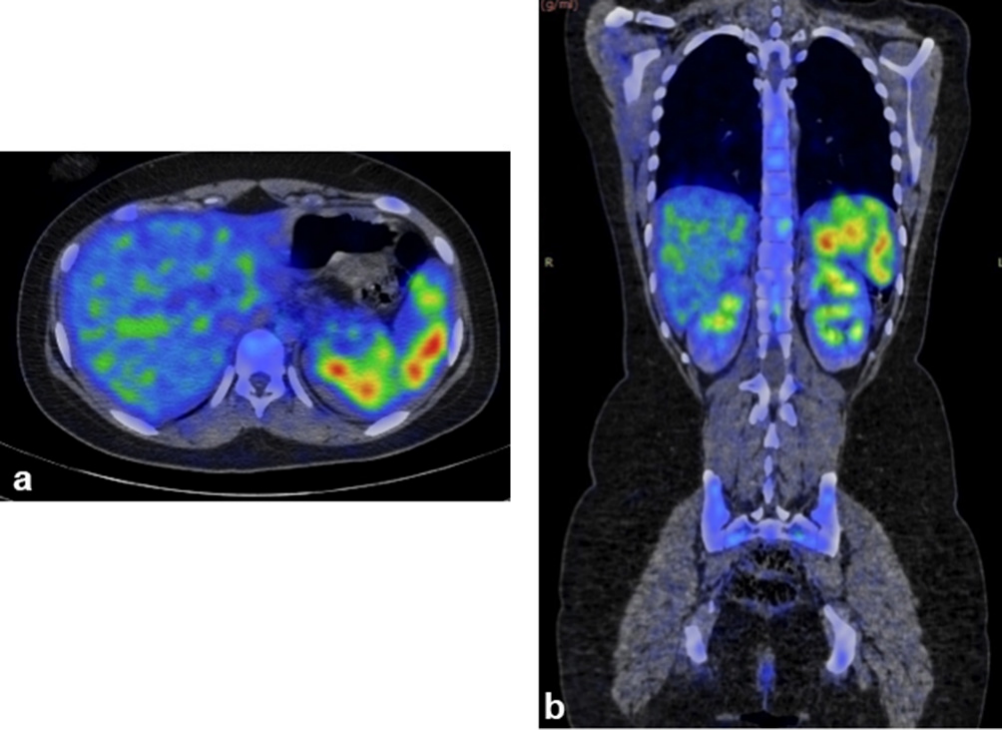
Fused fluorodeoxyglucose-positron emission tomography-CT (axial and coronal) images showing multiple focal fluorodeoxyglucose-avid nodular lesions in spleen.

**Figure 4.**
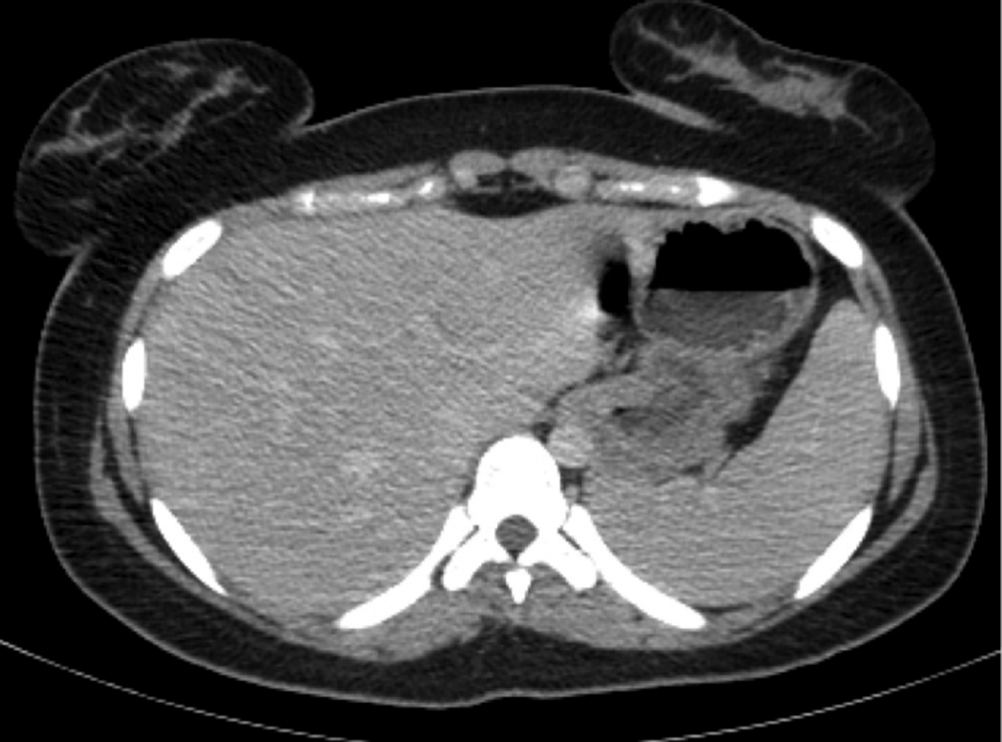
Corresponding contrast-enhanced CT image (axial section at the level of liver and spleen) showing normal splenic parenchyma without any focal nodular lesions.

**Figure 5. MR:**
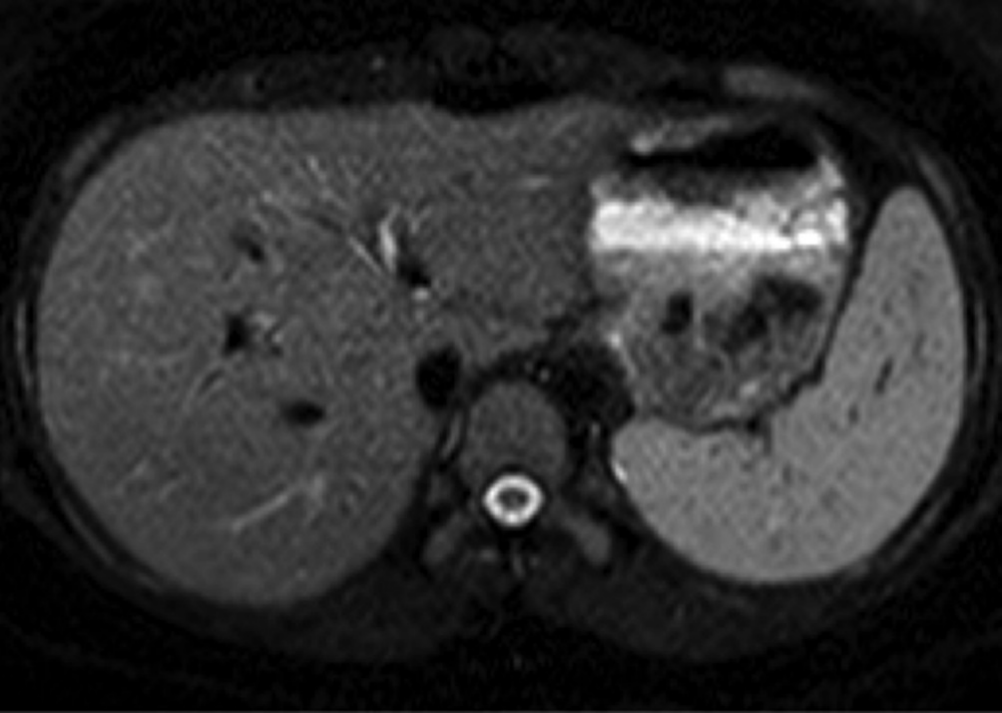
images at the level of liver and spleen not showing any focal lesions in spleen.

A 17-year-old female presented to the physician with a 4 weeks history of fever, predominantly with evening rise of temperature. The temperature fluctuated between 37.7 and 38.8°C. She had no history of cough, haemoptysis or significant loss of weight. Neither a previous history of TB nor a recent exposure to TB was evident. Physical examination of the patient did not reveal any positive information as there was neither hepatosplenomegaly nor lymphadenopathy. Routine haematological investigations were found be largely within normal limits except for elevated C-reactive protein (39 mg l^–1^) and mildly elevated gamma-glutamyl transferase (56 Ul^–1^). Her haemoglobin was 11 g dl^–1^, with slightly reduced mean corpuscular volume, mean corpuscular haemoglobin and mean corpuscular haemoglobin concentration. The investigations for various types of fevers, including Widal test for typhoid/ paratyphoid, malarial parasite, leptospira immunoglobulin M antibodies, Weil Felix test for rickettsial infection, urine test and sputum and blood culture were non-contributing. She tested negative for retrovirus infection. No significant pathology was identified on her chest radiograph. Abdominal ultrasound scan was unremarkable (Figure 1). Finally, an 18-fluorodeoxyglucose (FDG)-positron emission tomography (PET)-CT scan was performed, which demonstrated multiple focal areas of abnormally high FDG uptake within the spleen (Figures 2, 3), which were not identifiable on CT scan images (non-contrast CT and contrast-enhanced images) (Figure 4). No other focal abnormalities were detected anywhere else. MRI of the abdomen was performed for further characterization of the splenic lesions. However, MRI abdomen, including diffusion-weighted images, did not show any nodules in the spleen (Figure 5). To avoid splenectomy for histopathological diagnosis, biopsy of the splenic lesions was planned, after ensuring that the coagulation profile was normal. True–cut biopsy was done by placement of a coaxial needle into one of the lesions under CT scan guidance, after carefully analysing the PET images.

**Figure 6.**
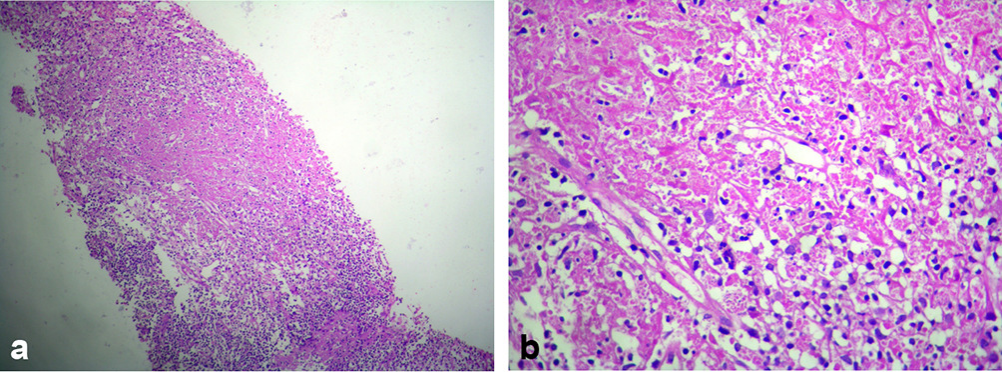
(a) Haematoxylin and eosin staining, 10×; (b) haematoxylin and eosin staining, 40×: Splenic biopsy showing caseating necrosis surrounded by rim of histiocytes and lymphocytes with some epithelioid transformation, along with scanty karyorrhectic debris.

Necrotizing granulomatous lesions suggestive of TB were identified on histopathology, as described in Figure 6a,b. The patient was started on antitubercular therapy for 9 months. The fever resolved by 3 weeks after the beginning of the treatment.

## Discussion

Extrapulmonary TB comprises 15–20% of all cases of TB. Abdominal TB is even more uncommon, and accounts for only 3%.^4^ Isolated splenic TB is rarely seen, that too in an immunocompetent person. In our extensive literature review, we could not find any definite reported incidence or prevalence of splenic TB. Splenic TB has been described in the immunocompromised, in patients with sickle cell disease, previous trauma, as a part of disseminated TB and in the setting of contiguous spread from pancreas.^2,4^ Our patient did not have any of these predisposing conditions and had no history of exposure to TB.

Splenic TB may be seen as an isolated splenic lesion, multiple splenic nodules, abscesses or hypersplenism. Splenic rupture is a rare complication of TB.^4^

Diagnosis of isolated splenic TB is certainly difficult. Many splenic lesions seen on imaging are nodular in morphology and differential diagnosis of abscesses, fungal infectious abscesses, sarcoidosis, lymphomas and metastasis needs to be considered in the context of pyrexia of unknown origin (PUO).^4–6^ Many patterns such as nodular, pseudotumour and ovoid appearances have been described in TB of spleen on CT scan. Five types of splenic TB can be identified based on pathomorphological appearances including miliary TB, nodular TB, tuberculous spleen abscess, calcific TB and mixed type TB.^7^ However, biopsy is the only way of confirmation. Ultrasound abdomen is the initial mode of imaging to detect the presence of splenic lesions. CT scan is the next modality to look for splenic lesions and also to detect concurrent lesions in the chest and other parts of the abdomen.

However, no lesions were detected on ultrasound in our patient. FDG-PET CT demonstrated the hypermetabolic lesions in the spleen. Contrast-enhanced diagnostic CT scan (performed at our institution as a part of the PET CT protocol for PUO) did not reveal any lesions at the foci of abnormal hypermetabolic activity on FDG-PET. Moreover, MRI including diffusion-weighed imaging, which was performed to further characterize the lesions, did not reveal any lesions in the spleen. This confirms the importance of FDG-PET imaging in detecting those lesions that may be undetected on other modalities. PUO is one of the non-oncological indications of whole-body FDG-PET CT. Its high sensitivity in detecting malignant lesions, infections and other inflammatory processes makes it an important tool in the investigation of a patient with PUO.^8,9^ Increased FDG uptake in active TB can be seen in diverse anatomical locations, mimicking malignant processes. FDG-PET CT does not demonstrate a characteristic pattern to differentiate between benign and malignant lesions.^8^

Dual time point imaging studies have documented the value of additional delayed images, obtained 90–120 min after FDG injection, in differentiating benign from malignant lesions. On delayed images, inflammatory lesions are reported to demonstrate increased FDG washout, whereas cancerous lesions usually exhibit further accumulation of tracer.^8,10^ Another advantage of FDG-PET CT is that it can also identify lesions at other unsuspected sites, the accessibility of which (for biopsy) may be easier. Splenic biopsy has a complication rate of 1.5–13%. Thus, splenic biopsy may be avoided if multiple other sites are involved.^6^ Also, an FDG-PET CT-guided biopsy may help in difficult situations where lesion identification on other modalities may be difficult or impossible.^11^

As in other extrapulmonary manifestations, patients with splenic TB are treated with anti-TB drugs. A full 12-month regime treatment is necessary. Splenectomy may be indicated when an abscess is present, if biopsy specimens are non- diagnostic or when the patient is not responding to treatment.^4^ Our patient has had remission of fever within 3 weeks and has not shown any clinical recurrence till now.

Isoniazid, rifampicin and pyrazinamide have been labelled with carbon-11 and the biodistribution of the labelled drug has been determined in baboons using PET.^12^ Thus future prospects of PET CT with labelling of antituberculous drugs such as isoniazid and rifampicin with positron emitting isotopes may result in the development of TB-specific PET radiopharmaceuticals.^8^ Further studies are needed in this regard.

## Conclusions

To our knowledge, this is the first case report in the English literature where isolated splenic TB is detected only on a functional study such as FDG-PET and not seen on any other morphological imaging studies.

## Learning points

1. TB should be considered as a differential diagnosis in any patient with fever, more so in the developing countries and in immigrants in the developed countries.
2. TB can present with atypical imaging findings and can be isolated to the spleen.
3. FDG-PET can add value in identifying lesions, which otherwise can be missed on other imaging modalities.
4. Combining FDG -PET with a proper diagnostic CT protocol can be suggested as a one-stop investigation for the evaluation of a patient with PUO.

## Consent

Informed consent could not be obtained despite exhaustive attempts to contact the patient after we decided to publish the case report. However, patient anonymity has been maintained.
